# Typhoid Fever; Its Antiseptic Treatment in Twenty-Five Consecutive Cases

**Published:** 1903-02

**Authors:** James Billingslea

**Affiliations:** Baltimore, Md.


### TYPHOID FEVER; ITS ANTISEPTIC TREATMENT IN TWENTY-FIVE CONSECUTIVE CASES.

By JAMES BILLINGSLEA, M.D.,

BALTIMORE, MD.

So much has been written on the subject of typhoid fever, and particularly in regard to its treatment, that it would seem almost impossible to add anything new; yet my experience during the past year, extending over a large number of cases, has been such that I feel that no apology is needed for laying some of its results before the profession.

The ubiquity of this dread disease, its mysterious and ghost-like mode of entry into a community, its almost certain outbreak in large camps, its unaccountable appearance in isolated spots, the frequent difficulties in its clinical diagnosis, its liability to serious complications and troublesome sequelæ, and its demoralizing effect upon the organism for some time after convalescence, coupled with a perfect scourge of mortality until at least quite recent times, render typhoid cases one of the most anxious cares that fall to the lot of the physician.

It is needless to do more than summarize the chief points of the disease, so well known are they. They consist of a general infection caused by Eberth's bacillus typhosus, and characterized, at any rate in the later stage, by hyperplasia and ulceration of the intestinal lymph follicles, swelling of the mesenteric glands and spleen, and parenchymatous changes in the organs. Yet while these lesions are fairly constant there are cases in which the local changes are very slight, or even altogether absent, and yet again others in which the poison shows itself localized in the lungs, spleen, kidneys, or cerebro-spinal system. Indeed Wasdin (1) has recently asserted, and certainly with considerable weight of evidence, that the primary route of infection is usually through the respiratory passages, and is evidenced by a smaller or greater area of subacute bronchitis, commonly localized to one lung. From this pulmonary atrium the bacillus enters the blood stream, (2) whereby it is deposited in the various other organs, spleen, kidneys, intestinal glands, etc., producing thereby the secondary series of symptoms so well known in the second and third weeks of the disease.

As bearing on this view the increasing evidence in favor of an air-borne infection (3) as one mode of entrance calls for mention. There seems to be a general tendency, when one mode of infection has been established, to resent any attempt to claim a division of honors for another. It is indisputable that the malarial mosquito is the host of the parasite and a mode of infection of man therewith; but that does not prevent the possibility of there being other modes of infection. Indeed, Dr. Dodd (4), writing from Talas in Asia Minor, told of the commonness of malaria in that place, four thousand feet above the sea level, with dry climate, rocky soil, scanty vegetation and absolutely no mosquitoes, although ten years previously malaria had been entirely unknown there. In like manner the fact that typhoid may be water-borne, the certainty with which it has been traced at times to milk and other food products, by no means preclude the possibility of an air-borne origin. Indeed Col. Quill, R.A.M.C., (5) has reported an outbreak in Ceylon among the guards of Boer prisoners, and imported from Africa by the latter, although the water-supply was brought three miles through underground iron pipes and filtered in Berkefield filters, and no fresh milk or uncooked fruit or vegetables were allowed. But the prisoners' latrines were very close to the guards' quarters, flies abounded, and dust storms were prevalent.

These considerations are important in regard to the matter of treatment. The expectant, do-nothing treatment, in vogue some time back, is practically abandoned, owing to the disastrous records it bore. The fact that typhoid is essentially a blood-poisoning clearly indicates an antiseptic or eliminative, or both.

Various antiseptics have been tried aud vaunted. Erb (6) particularly eulogized quinine, but it does not do well in complications or mixed infections, nor does it shorten the course of the disease. Bardet (7) has recommended carbon-disulphid water. Other antiseptics that have been tried are baptisia tinctoria, bismuth, beta-naphtol, boric acid, chlorine, salicylates, sulphurous acid, salol and turpentine. What is really needed, however, for internal antisepsis to be of any effect, is an antiseptic sufficiently powerful—say as powerful as 1:1000 bichloride—and yet innocuous to the animal organism.

Eliminative measures have been freely tried, and one of the most potent agents that we have to-day, Brand's method of cold bathing, (8) owes its merits largely to its eliminative value. But even the undoubted value of Brand's method does not militate against the importance of an antiseptic plan if an effective one were forthcoming. There are cases where Brand's method cannot well be applied, viz., often in private houses, in out-of-the-way country districts, or in wide-spread epidemics where the necessary trained attendance cannot be forthcoming. There are other cases, again, where it is unsuitable—*e. g.*, in the very old or feeble, and where the heart is very weak, or where terror might do greater harm than good. Finally, where none of these objections exist,

Brand's method can be carried out in conjunction with the antiseptic, with increased value to both.

There is a further value to be looked for from an effective antiseptic treatment. Recent researches (9) have disclosed the existence of various forms of clinical typhoid, which, however, fail to fulfill the bacteriological test. It is probable that these cases belong to two groups of infection whose symptoms closely resemble typhoid, but differ in some particulars and especially as regards Widal's reaction. These are infections due to groups of bacilli intermediate between the colon and typhoid bacilli, and subdivided into parcolon or paratyphoid, as they approximate to one or the other. It is tolerably certain, however, that, given a really effective antiseptic, for clinical purposes all distinction between these groups might be ignored. An antiseptic as powerful as bichloride, but quite innocuous, would most probably prove of equal service, whatever the nature of the bacillary infection.

Such an antiseptic seems to exist in the case of acetozone (benzoyl-acetyl-peroxide), which comes to us with the indorsement of Professors Freer and Novy, of Ann Arbor University, Mich. Wasdin, also, has recorded in the paper before referred to twenty-four cases treated with this drug, with a speedy subsidence of fever, a small proportion of complications, and a material lessening of the duration of the disease. Now, while I have always been an advocate of conservative treatment, and have made it my aim to "hasten slowly," the foregoing facts have compelled me, seeing as I do a large number of typhoid cases, to give it a fair trial. During the past year I have treated with acetozone in private and hospital practice twenty-five cases of typhoid fever, and the results have certainly surpassed my expectations, not a single death having occurred. In no case was there delirium or any other complication; no tympanitis or diarrhea, and of course no hemorrhages. Acetozone seemed to render the system immune to the typhoid toxin, so that if the patient came under treatment at the outset, the disease seemed to be jugulated; while, when it did not come under observation until the disease was well established—say within the first seven days—it was yet materially shortened by about one week. A marked influence was exerted on the temperature, a point of much significance, which requires no enlarging upon. It has been my practice to have my cases of typhoid diagnosis submitted to the Board of Health for confirmation, and certificates from the board will prove conclusively that the favorable course these cases have run has not been due to an error in diagnosis. For my own part I feel no hesitation in ascribing the results to the treatment adopted, which was essentially that described recently by Dr. Wasdin, and also practiced at the Cook County Hospital. The first thing is to clear the bowels thoroughly by a good dose of calomel, which may be administered in one large dose or in frequently repeated small doses, as the physician thinks best. Liquid diet is prescribed, and cold sponge or tub baths, according to the generally adopted methods may be ordered as occasion calls for them.

The special treatment consists in dissolving 15 to 20 grains of acetozone powder (equal to 7½ or 10 grains of the benzoyl-acetyl-peroxide) in a quart of water and giving the patient this amount to drink in the twenty-four hours both in water and in milk, diluting the milk one part of acetozone solution to three parts of milk. The action of the drug will be materially aided by administering a mild saline laxative, say sodium phosphate or magnesium sulphate, every other day. In addition to the advantages of the treatment already enunciated, I may add that I have found that the feces soon lose the disagreeable odor that commonly characterizes typhoid stools; that while cold baths may be used as required, they are required to a much smaller extent than with other treatments; that the nurses universally affirm that they find the patients under this line of treatment much easier to care for and to manage; and that no evil results have, so far, been noted from the use of acetozone. On all counts, therefore, I sincerely trust that the profession will accord to this treatment a fair and unprejudiced trial.

(1) *American Medicine*, Feb. 8, 1902.

(2) Cole, *Johns Hopkins Hos. Bull.*, July, 1901; Courmont, *Lyons Medical*, Jan. 26, 1902.

(3) Victor C. Vaughan, *Jour. Amer. Med. Ass'n*, April 19, 1902.

(4) *Medical Record*, Oct. 8, 1898.

(5) *Brit. Med. Journ.*, Feb. 15, 1902.

(6) *Therapia d. Gegenu*, 1901, XLII., p. 1.

(7) Bardet, Rev., *Medicale*, June 19, 1902.

(8) For a good account of Brand's and other hydropathic measures see Russell Bellamy, "Modern Methods in the Management of Typhoid Fever, etc." *New York Medical Journal*, Sept. 7, 1901, p. 433.

(9) For summary, see Warren Coleman, "Types of Infection Produced in Man by Intermediate Members of the Typhoid-Colon Group of Bacilli," *Amer. Med.*, Sept. 27, 1902, p. 498. Also Meltzer, "Paratyphoid," *N. Y. Med. Jour.*, Jan. 25, 1902, p. 138.

---

### THE COCAINE HABIT AMONG THE NEGROES.

The cocaine habit among the negroes of the South has of late become alarmingly prevalent. The negroes of New Orleans found that the drug enabled them to perform more easily the extraordinarily severe work of loading and unloading steamboats. The high wages offered by the Mississippi river steamboat owners for the unloading of cargoes is said to have first prompted the negroes to use cocaine as a stimulant. They soon became aware that they could work for seventy hours at a stretch in rain, in cold, and in heat when under the influence of cocaine. This state of affairs is one that certainly calls for the attention of the authorities, and strenuous efforts should be put forth to stop the sale of this drug to the ignorant negro roustabouts of the South.—*Med. Age.*

